# The relationship of lipoprotein-associated phospholipase A2 activity with the seriousness of coronary artery disease

**DOI:** 10.1186/s12872-020-01580-4

**Published:** 2020-06-16

**Authors:** Hao Zhang, Yang Gao, Dan Wu, Dingguo Zhang

**Affiliations:** 1 Department of Cardiology, The Affiliated Hospital of Yangzhou University, 41# Taizhou road, Yangzhou, 225000 Jiangsu Province China; 2 Department of Cardiology, First Affiliated Hospital of Nanjing Medical University, 300# Guangzhou road, Nanjing, 210029 Jiangsu Province China

**Keywords:** Lipoprotein-associated phospholipase A2, Cardiovascular disease, Coronary artery diseases, Coronary angiography

## Abstract

**Background:**

The level of lipoprotein-associated phospholipase A2 (LP-PLA2) in serum is independently correlated to coronary artery diseases (CAD). The aim of the study was to determine whether LP-PLA2 activity is positively associated with the seriousness of CAD.

**Methods:**

Amount to 1056 patients suspected of having CAD underwent coronary angiography (CAG) to determine the seriousness of CAD. According to the amount of diseased coronary branches, the 1056 patients were split into three groups: single-vessel stenosis group, multiple-vessels stenosis group (> or = 2 diseased coronary branches),and control group (no diseased coronary branches). According to CAG results, electrocardiography, cardiac biomarker, and clinical presentation, all patients were split into four groups: acute myocardial infarction (AMI), unstable angina (UA), stable angina (SA), and control groups (excluding CAD). The activity of LP-PLA2 was compared statistically among the subgroups. Receiver operating characteristic analysis was applied to investigate the role of LP-PLA2 in evaluating the presence and seriousness of CAD.

**Results:**

The level of LP-PLA2 increased in line with the number of diseased coronary branches. The levels of LP-PLA2 in the AMI and UA groups were observably higher when compared with the control and SA groups. LP-PLA2 had 75.6% sensitivity and 67.3% specificity for recognizing CAD, and 53.0% sensitivity and 80.3% specificity for recognizing severe coronary artery lesions.

**Conclusion:**

The activity of LP-PLA2 is positively correlated to the seriousness of CAD.

## Background

Coronary artery diseases (CAD) remain the main cause of mortality and functional impairment all over the world, in spite of huge progress on medical therapeutics in recent decades. It is widely acknowledged that inflammatory reactions contribute considerably to the production of atherosclerosis, from original atherosclerotic plaque to subsequent destabilization and eventual rupture [1]. Several previous researches have suggested that numerous inflammatory biomarkers are relevant to cardiovascular diseases (CVD); one of the most typical of these is C-reactive protein (CRP) [2, 3].

LP-PLA2 is an enzyme produced mainly by neutrophils and macrophages from atherosclerotic plaques, which are then transferred to the bloodstream via high-density lipoprotein-cholesterol (HDL-C) as well as low-density lipoprotein-cholesterol (LDL-C) [4, 5]. Plenty of evidences have indicated that, as an important biomarker of inflammation, Lp-PLA2 is an independent risk factor for CVD [6]. Several researches have demonstrated that higher plasma levels of LP-PLA2 activity further increase the risk of cardiovascular events, for instance ischemic stroke and myocardial infarction [7]. However, the correlation between the level of LP-PLA2 and CVD has not necessarily been confirmed in other research [7–9]. Besides, two recent clinical studies suggested that darapladib, as a selective LP-PLA2 antagonist, may not improve clinical outcomes in patients with CAD [10, 11]. The results of previous research appear to be contradictory.

In view of these conflicting findings, we carried out a targeted clinical research to observe whether increased activity of LP-PLA2 is positively correlate to the seriousness of CAD. The findings of our study would provide meaningful information to confirm whether the risk degree of CAD could be predicted independently by LP-PLA2 level.

## Methods

### Patients and protocols

All the patients were consecutively recruited from December 2016 to June 2019 in the Department of Cardiology, First Affiliated Hospital of Nanjing Medical University. All the patients gave informed consent, and the Ethical Committee of Nanjing Medical University approved the research. All enrolled subjects underwent coronary angiography (CAG) to establish the severity of CAD. All patients underwent CAG in accordance with the requirements of coronary angiography guidelines [12]. According to the number of diseased coronary branches (≥50% stenosis), 1056 patients were split into three groups: single-vessel stenosis (SVS) group, multiple-vessel stenosis (MVS) group (≥2 diseased coronary branches), and control group (< 50% stenosis). According to the CAG results, cardiac biomarker, electrocardiography, as well as clinical presentation, subjects were split into four groups: AMI, UA, SA, and control groups (excluding CAD).

The demographic characteristics of all patients, including age, gender, smoking status, and clinical characteristics (such as diabetes mellitus and hypertension), were obtained via questionnaire [13]. Blood samples were collected from these subjects for various blood index analyses. Fasting blood glucose (FBG), lipoprotein(a) (Lp(a)), total cholesterol (TC), high-density lipoprotein-cholesterol (HDL-C), low-density lipoprotein-cholesterol (LDL-C) and triglyceride (TG) were tested using fasting venous blood on day 1 of hospital admission. The measurement of blood lipid is carried out by the automatic biochemical analyzer (Tokyo Medical 1024i, Japan). Determination of the plasma level of LP-PLA2 was done using an enzyme-linked immune-absorbent assay (ELISA) kit (commercially bought from Nanjing Norman Biological Technology Co. Ltd., China). The process of the testing the level of LP-PLA2 was conducted according to the manufacturer’s instructions to maintain quality control. Meanwhile, three independent processes were conducted in duplicate, and the mean value was calculated. Receiver operating characteristic (ROC) curves were drawn, and areas under the curve (AUC) were compared to assess the cut-off values of LP-PLA2 to judge the presence and severity of CAD. The severity of CAD is mainly judged by comparing single-vessel lesions with multiple-vessel lesions.

### Analysis of data and statistics

Continuous variables that satisfied the normal distribution were shown as means ± standard deviation (SD). All measured data were analyzed to compare differences between groups, using one-way ANOVA and Student’s t-test. Data described as proportions were assessed with a Chi-square test. For non-normally distributed variables,we use the median and inter-quartile range to represent. Evaluation of the relationship between the seriousness of CAD and the plasma level of LP-PLA2 was performed using univariate and multivariate regression analyses. *P* values were two-sided, and P values≤0.05 were considered as statistically significant. All analyses were conducted using SPSS software package version 21.0 (Chicago, IL, USA).

## Results

In all, 1056 studied subjects underwent CAG. The results showed that the coronary artery of 294 patients were without significant stenosis (< 50%), who were regarded as the control group. Meanwhile, 762 patients were diagnosed as having coronary atherosclerotic heart disease (at least one coronary artery stenosis ≥50%); this group contained 366 patients with SVS and 396 with MVS. According to the severity of clinical presentation, all patients diagnosed as having CAD were divided into SA (*n* = 192), UA (*n* = 330), and AMI (*n* = 240) groups.

### Assessment of the relationship between the level of LP-PLA2 and degree of coronary artery stenosis

As shown in Table 1, participants diagnosed as being in the MVS group had several risk factors compared to the control group. Generally, participants with MVS were older. Their levels of serum Lp(a) and FBG were visibly higher, while the level of HDL-C was dramatically lower in patients with MVS in comparison to the controls. The basic data of the SVS group showed no significant difference compared with the controls and MVS group. This shows that those in the SVS group expressed an obviously high level of LP-PLA2 in comparison to the control group, while the MVS group also showed a apparent increase in expression of LP-PLA2. After adjusting for gender, age, smoking, hypertension, diabetes mellitus, FBG, Lp(a), TC, TG, LDL-C, and HDL-C, the level of LP-PLA2 expression was still independently associated with the degree of coronary artery stenosis, with an odd ratio (OR) of 1.011 (MVS versus control group, 95% confidence interval (CI) 1.006–1.017, *P* < 0.05).

**Table 1:** The relationship between the level of LP-PLA2 and degree of coronary artery stenosis

Group	Control	Single	Multiple	***P***
N	294	366	396	
Age (years)	60.63 ± 11.96	63.16 ± 12.88	65.23 ± 10.93*	0.113
Male(%)	204 (69.4)	306 (83.6)	282 (71.2)	0.237
Smoking(%)	84 (28.6)	150 (41.0)	162 (40.9)	0.315
Hypertension(%)	168 (57.1)	216 (59.0)	276 (69.7)	0.305
Diabetes (%)	54 (18.4)	96 (26.2)	120 (30.3)	0.345
FBG (mmol/L) ^a^	5.35 (4.39–8.56)	6.04 (5.32–10.25)	6.27 (4.28–10.45)*	0.204
Lp(a)(mmol/L) ^a^	230.39 (112.06–428.32)	262.12 (121.46–675.28)	346.55 (176.55–728.36)*	0.059
TC (mmol/L)	4.40 ± 1.13	4.20 ± 0.20	4.52 ± 1.28	0.291
TG (mmol/L)	1.52 ± 0.97	1.51 ± 0.87	1.78 ± 0.87	0.213
LDL-C (mmol/L)	2.82 ± 0.93	2.59 ± 0.84	2.88 ± 1.02	0.192
HDL-C (mmol/L)	1.17 ± 0.28	1.09 ± 0.23	1.04 ± 0.26 *	0.027
Lp-PLA2(ng/ml) ^a^	172.83 (92.35–296.42)	230.95 (115.69–406.35)*	289.24 (132.11–430.21)*^#^	< 0.001

^a^indicated median and inter-quartile range, FBG Fasting blood glucose, *Lp(a)* Lipoprotein(a), *TC* Total cholesterol, *TG* Triglyceride, *LDL-C* Low density lipoprotein-cholesterol, *HDL-C* High density lipoprotein-cholesterol, *Lp-PLA2* Lipoprotein associated-phospholipase A2. *P < 0.05 versus control group, ^#^*P* < 0.05 versus single group

### Assessment of the relationship between the level of LP-PLA2 and seriousness of clinical presentation

All studied subjects were split into four groups according to the seriousness of clinical presentation, as demonstrated in Table 2. The HDL-C level of the UA and AMI groups showed a apparent increase in comparison to the control group. In comparison to the SA and control groups, LP-PLA2 level was dramatically higher in the UA and AMI groups. No obvious difference were observed between the UA and AMI groups, while no obvious difference between the SA and control groups were observed. After adjusting for gender, age, smoking, hypertension, diabetes mellitus, FBG, Lp(a), TC, TG, LDL-C, and HDL-C, the level of LP-PLA2 expression was still independently associated with the seriousness of clinical presentation, with an OR of 1.014 (AMI versus control group, 95% CI 1.008–1.021, *P* < 0.05).

**Table 2:** The relationship between the level of LP-PLA2 and seriousness of clinical Presentation

Group	Control	SA	UA	AMI	***P***
N	294	192	330	240	
Age (years)	60.63 ± 11.96	66.00 ± 13.66	64.80 ± 10.99	62.05 ± 11.58	0.130
Male(%)	204 (69.4)	150 (78.1)	252 (76.4)	186 (77.5)	0.762
Smoking(%)	84 (28.6)	66 (34.3)	138 (41.8)	108 (45)	0.362
Hypertension(%)	168 (57.1)	120 (62.5)	252 (76.4)	120 (50)	0.051
Diabetes (%)	54 (18.4)	60 (31.2)	84 (25.5)	72 (30)	0.514
FBG (mmol/L)^a^	5.35 (4.39–8.56)	6.13 (4.69–10.36)	6.14 (4.72–10.38)	6.23 (4.56–10.42)	0.071
Lp(a) (mmol/L)^a^	230.39 (112.06–428.32)	282.16 (115.22–652.36)	308.20 (122.08–721.12)	322.03 (130.06–732.16)	0.390
TC (mmol/L)	4.40 ± 1.13	4.25 ± 1.13	4.31 ± 1.19	4.53 ± 1.15	0.724
TG (mmol/L)	1.52 ± 0.97	1.65 ± 0.96	1.64 ± 0.96	1.65 ± 0.69	0.782
LDL-C (mmol/L)	2.82 ± 0.93	2.65 ± 0.89	2.65 ± 1.02	2.94 ± 0.88	0.432
HDL-C (mmol/L)	1.17 ± 0.28	1.11 ± 0.22	1.05 ± 0.27*	1.04 ± 0.24*	0.056
Lp-PLA2(ng/ml) ^a^	172.83 (92.35–296.42)	193.84 (102.35–372.35)	263.95 (172.32–396.45)*^&^	311.45 (185.24–412.35)*^&^	< 0.001

^a^indicated median and inter-quartile range, *P < 0.05 versus control group, ^&^*P* < 0.05 versus SA group

### Assessment of the presence and severity of CAD by cut-off values of LP-PLA2

**Fig. 1:**
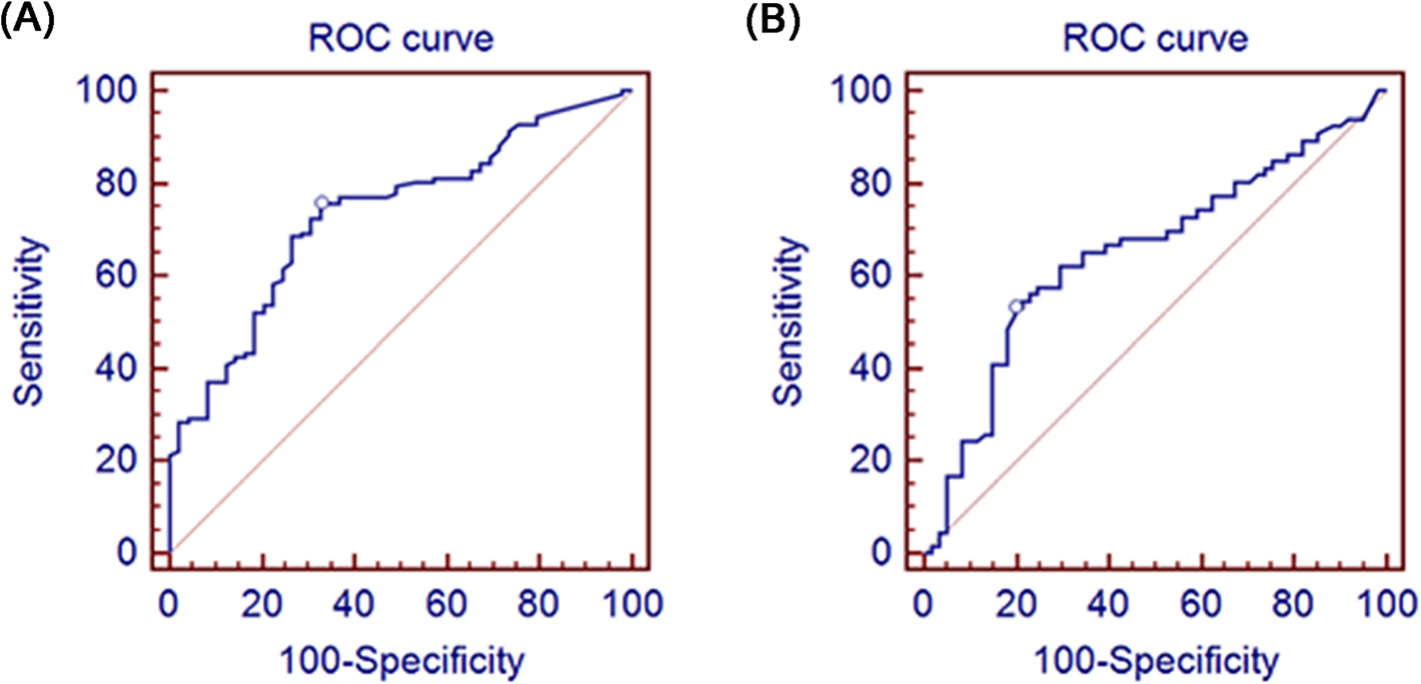
ROC analysis results for LP-PLA2 in assessing the presence and severity of CAD. **a** ROC analysis results for LP-PLA2 in assessing the presence of CAD; **b** ROC analysis results for LP-PLA2 in assessing the severity of CAD

The ROC curve analysis demonstrated the AUC of LP-PLA2 in judging the presence and severity of CAD (Fig. 1). The AUC were 0.734 (Fig. 1a) and 0.652 (Fig. 1b), respectively. LP-PLA2 had 75.6% sensitivity and 67.3% specificity for recognizing CAD, and 53.0% sensitivity and 80.3% specificity for recognizing severe CAD.

## Discussion

The previous study has confirmed that the development of CAD is closely related to the inflammatory response, while the dynamic process of CAD is resulted from the interaction between the inflammatory response and vascular endothelial dysfunction. LP-PLA2 is an enzyme mainly separated from atherosclerotic plaques, which was produced inflammatory cells and circulates in the bloodstream. Due to its important effects on atherosclerosis [14, 15], LP-PLA2 was considered a promising biomarker for the evaluation of CVD risk. Evidence from basic and clinical studies also indicated that decreased LP-PLA2 level played important role in slowing atherosclerosis and cutting down the occurrence of CAD [16, 17].

Some studies have shown that the increase of LP-PLA2 level is closely correlated to the incidence of acute coronary syndrome. At the same time, LP-PLA2 can also predict the recurrence of angina, heart failure, myocardial infarction and other cardiovascular events [18]. And there is evidence that LP-PLA2 has a significant clinical predictive value for the occurrence and development of CAD [19]. Therefore, it is reasonable to speculate that elevated plasma level of LP-PLA2 might contribute to accelerating the progression of coronary artery stenosis as well as atherosclerotic plaque rupture. Results of our cross-sectional research also support the hypothesis.

Previous studies from Shahar Lavi and institution have hinted that the production of LP-PLA2 is correlated to endothelial dysfunction as well as early coronary atherosclerosis [20]. Different from previous studies, our study is the first to explore the correlation between phospholipase A2 and the amount of coronary artery lesions. The results demonstrate that LP-PLA2 level in the SVS and MVS groups was obviously increased compared to the control group. Therefore, we speculated that the more serious the endothelial dysfunction and coronary atherosclerosis, the higher the plasma LP-PLA2 level would be. Although it is regrettable that we did not study the endothelial function of each group, several clinical and basic experiments have confirmed the positive correlation between LP-PLA2 and endothelial dysfunction. Research from Iwase M showed that, in addition to endothelial-associated mechanisms, oxidized fatty acid and hemolytic lecithin play vital roles in the development of atherosclerotic plaques as well [21]. In general, we believe that LP-PLA2 could accelerate atherosclerotic plaque development.

LP-PLA2 can provide important value for the prevention as well as treatment of cardiovascular and cerebrovascular diseases. Data from Berger JS show that an LP-PLA2 inhibitor can significantly ameliorate the occurrence of cardiovascular events compared with a placebo [22]. Daida H also believed that decreasing the level of plasma LP-PLA2 could not only delay the process of atherosclerosis but also decrease the occurrence of cardiovascular events [17]. However, data from the latest clinical trials suggested that using the LP-PLA2 inhibitor darapladib treatment may not decrease the occurrence of CAD [11, 23], which seems to contradict previous research. Although in our research, the plasma level of LP-PLA2 in the AMI and UA groups was remarkably higher than that in the control group after adjustment of several basic data, and the LP-PLA2 level gradually rose with the number of coronary artery lesions, we think this is due to the continuous release of LP-PLA2 from unstable and ruptured plaques. All this indicates that detecting LP-PLA2 activity is useful not only for judging the severity of CAD but also for estimating the severity of coronary atherosclerosis, which is consistent with data from Blankenberg [24]. For the first time, we used ROC curve to assess the diagnostic value of LP-PLA2 for CAD. ROC curve analysis reveals that LP-PLA2 can be used not only to evaluate the occurrence of CAD but also to judge its severity. We speculate that the risk of cardiovascular events will increase with the rising level of LP-PLA2 in plasma.

There are some limitations in this study. First, the time of research was relatively short and the number of cases relatively small, so it still needs a major case supplement. Besides, we did not exclude patients who need to take hypolipidemic drugs and aspirin before drawing blood, which may have had an impact on the results. However, Anping Cai thought that statins do not affect the correlation between LP-PLA2 level and the seriousness of CAD [13]. Finally, there may be cases of that certain patients with non-significant CAD (small lesions) develop unstable angina due to plaque instability, this may have some influence on the research results, however, we believe that the impact is limited.

## Conclusions

In conclusion, as a relatively new inflammatory marker, the effect of LP-PLA2 in atherosclerosis is still controversial, but more and more evidence indicates that it plays a an important role in the occurrence and development of coronary atherosclerosis. The current study also shows that LP-PLA2 activity is positively correlated to the seriousness of CAD, and this can provide a strong basis for predicting the occurrence and prognosis of CAD, at the same time, it may provide a new way to treat CAD in the future.

## Data Availability

The datasets used and/or analysed during the current study are available from the corresponding author on reasonable request.

## Abbreviations

CAD: Coronary artery diseases
CAG: Coronary angiography
SA: Stable angina
UA: Unstable angina
AMI: Acute myocardial infarction
CVD: Cardiovascular diseases
CRP: C-reactive protein
LP-PLA2: Lipoprotein-associated phospholipase A2
SVS: Single-vessel stenosis
MVS: Multiple-vessel stenosis
FBG: Fasting blood glucose
Lp(a): Lipoprotein(a)
TC: Total cholesterol
TG: Triglyceride
LDL-C: Low-density lipoprotein-cholesterol
HDL-C: High-density lipoprotein-cholesterol
ELISA: Enzyme-linked immune-absorbent assay
ROC: Receiver operating characteristic
AUC: Areas under the curve
OR: Odd ratio
